# Gene Expression of Glucose Transporter 1 (GLUT1), Hexokinase 1 and Hexokinase 2 in Gastroenteropancreatic Neuroendocrine Tumors: Correlation with F-18-fluorodeoxyglucose Positron Emission Tomography and Cellular Proliferation

**DOI:** 10.3390/diagnostics3040372

**Published:** 2013-10-29

**Authors:** Tina Binderup, Ulrich Peter Knigge, Birgitte Federspiel, Peter Sommer, Jane Preuss Hasselby, Annika Loft, Andreas Kjaer

**Affiliations:** 1 Department of Clinical Physiology, Nuclear Medicine and PET, Copenhagen University Hospital, Rigshospitalet & Cluster for Molecular Imaging, University of Copenhagen, Blegdamsvej 9, 2100 Copenhagen O, Denmark; E-Mails: Annika.loft.jakobsen@regionh.dk (A.L.); akjaer@sund.ku.dk (A.K); 2 Department of Surgical Gastroenterology, Copenhagen University Hospital, Rigshospitalet, Blegdamsvej 9, 2100 Copenhagen O, Denmark; E-Mail: Ulrich.peter.knigge@regionh.dk; 3 Department of Pathology, Copenhagen University Hospital, Rigshospitalet, Blegdamsvej 9, 2100 Copenhagen O, Denmark; E-Mails: birgitte.federspiel.01@regionh.dk (B.F.); jane.preuss.hasselby@regionh.dk (J.P.H); 4 Department of Urology, Copenhagen University Hospital, Rigshospitalet, Blegdamsvej 9, 2100 Copenhagen O, Denmark; E-Mail: peter.sommer@regionh.dk

**Keywords:** neuroendocrine tumors, glucose, gene expression, imaging, FDG-PET, proliferation index

## Abstract

Neoplastic tissue exhibits high glucose utilization and over-expression of glucose transporters (GLUTs) and hexokinases (HKs), which can be imaged by ^18^F-Fluorodeoxyglucose-positron emission tomography (FDG-PET). The aim of the present study was to investigate the expression of glycolysis-associated genes and to compare this with FDG-PET imaging as well as with the cellular proliferation index in two cancer entities with different malignant potential. Using real-time PCR, gene expression of GLUT1, HK1 and HK2 were studied in 34 neuroendocrine tumors (NETs) in comparison with 14 colorectal adenocarcinomas (CRAs). The Ki67 proliferation index and, when available, FDG-PET imaging was compared with gene expression. Overexpression of GLUT1 gene expression was less frequent in NETs (38%) compared to CRAs (86%), *P* = 0.004. HK1 was overexpressed in 41% and 71% of NETs and CRAs, respectively (*P* = 0.111) and HK2 was overexpressed in 50% and 64% of NETs and CRAs, respectively (*P* = 0.53). There was a significant correlation between the Ki67 proliferation index and GLUT1 gene expression for the NETs (R = 0.34, *P* = 0.047), but no correlation with the hexokinases. FDG-PET identified foci in significantly fewer NETs (36%) than CRAs (86%), (*P* = 0.04). The gene expression results, with less frequent GLUT1 and HK1 upregulation in NETs, confirmed the lower metabolic activity of NETs compared to the more aggressive CRAs. In accordance with this, fewer NETs were FDG-PET positive compared to CRA tumors and FDG uptake correlated with GLUT1 gene expression.

## 1. Introduction

Neoplastic tissue exhibits high glucose utilization. F-18-Fluorodeoxyglucose-positron emission tomography (FDG-PET) is widely used for imaging of this increased metabolic activity characteristic of malignant cells [1]. In comparison with other neoplasms, the majority of neuroendocrine tumors (NETs) are slow growing with low proliferation index and accordingly a good prognosis. The aggressiveness and thereof treatment of the NETs is mainly determined by immune-histochemical (IHC) staining for the proliferation factor, Ki67 [2]. Approximately 85% of NETs are low-malignant belonging to WHO class I and II according to the WHO 2010 classification of NETs with Ki67 proliferation index below 20% [3]. Therefore, FDG-PET seems to have a lower diagnostic sensitivity for these gastrointestinal tumors compared to e.g., the more aggressive colorectal adenocarcinomas [4]. 

The uptake of the glucose analogue, FDG, through the cell membrane is mediated by facilitative glucose transporters (GLUTs). Hexokinases mediates the subsequent intracellular phosphorylation [5]. In contrast to D-glucose, intracellular dephosphorylation of FDG by glucose-6-phosphatase is very limited, and FDG gets trapped and accumulates within the cell. This trapping mechanism is crucial for imaging with FDG-PET. Of the many subtypes of glucose transporters and hexokinases, the GLUT1 subtype is considered to be the glucose transporter mainly upregulated in cancer cells [6,7] and HK1 and HK2 are the hexokinase subtypes most frequently found overexpressed in cancer cells [8]. 

In agreement with the high diagnostic sensitivity of FDG-PET for colorectal adenocarcinomas (CRAs) [9,10], there is strong evidence for a high expression level of glycolysis associated genes in CRA [5,11]. Studies in NETs of the lung have shown a correlation between the expression level of GLUT1 and FDG uptake [12]. Knowledge regarding the expression level of glycolytic genes in gastroenteropancreatic NETs is however sparse. Whether the suitability of FDG-PET imaging can be explained by difference in aggressiveness and differences in gene expression levels of the glycolysis-associated genes GLUT1, HK1 or HK2 has yet to be determined.

The aim of the present study was to investigate whether the expression pattern of the glycolysis associated genes was different in NETs compared to the generally more aggressive CRAs, and whether the gene expression reflected the generally low-malignant characteristic of NETs. In addition, gene expression was correlated with proliferation index and FDG-PET imaging when available.

## 2. Experimental Section

### 2.1. Ethics Statement

The study was conducted in accordance with the declaration of Helsinki and approved by the regional scientific ethical committee of Copenhagen and Frederiksberg. Written informed consent was obtained from participants.

### 2.2. Patients/Subjects

Thirty-four patients (16 men and 18 women, mean age 54 (range 18–73 years)), diagnosed with NETs were enrolled in the study. Patients were enrolled in the study consecutively, when they were admitted to surgery for tumor resection. The histopathological diagnoses of the 34 patients were 11 ileal carcinoids, 9 pancreatico-duedenal NETs, 9 pheochromocytomas, 4 colonic NE carcinomas and 1 gastric NET, type 3. Patient characteristics are shown in Table 1. For comparison, 14 patients (7 men and 7 women, mean age 64 (range 33–77 years)) with liver metastases from colorectal adenocarcinomas (CRA) were enrolled, and the tissues examined from the CRA patients were in all cases from the liver metastases. 

For visualization of pathological foci, the accumulation of tracer in the tumors needs to exceed the tracer uptake in the surroundings. Accordingly, non-cancerous tissue collected from the resected specimens was used as calibrator of gene expression in both groups. 

The study was approved by the regional scientific ethical committee of Copenhagen and Frederiksberg and written informed consent was obtained from participants.

### 2.3. ^18^F-FDG-PET Imaging

Eleven patients in the NET group and 7 patients in the CRA group underwent ^18^F-FDG-PET/CT imaging prior to tumor resection according to our routine procedure [13]. PET/CT images were acquired 1 hour post-injection of 369–420 MBq 18F-FDG. All patients were instructed to fast for at least 6 h prior to the F-18-FDG injection. Blood glucose was measured prior to the FDG injection, and for all patients, the level was below 8 mmol/L.

The PET/CT scans were performed either by a GE Discovery LS PET/CT scanner or a Siemens Biograph 16 PET/CT scanner. Emission scan time was 3 min per bed position. The CT scans were performed as low-dose CT scans with 10 mA for minimization of the radiation burden. The CT data were used for attenuation correction of the PET data. The PET and low-dose CT images were reconstructed in all three axes and fused and analyzed on the GE eNTEGRA PET workstation and Siemens Leonardo workstation, respectively. The low-dose CT images were used as anatomical guides for localization of the pathological foci. 

**Table 1:** .Patient descriptions (neuroendocrine tumor (NET) group).

Tumor type	Site of sample collection ^a^	Metastatic disease	Ki67 index, %
Ileal carcinoid	Retro-peritoneal LN (m)	Yes	<2
Ileal carcinoid	Ileum (p)	Yes	<2
Ileal carcinoid	Ileum (p)	Yes	<2
Ileal carcinoid	Ileum (p)	Yes	<2
Ileal carcinoid	Ovary (m)	Yes	40
Ileal carcinoid	Retro-peritoneal LN (m)	Yes	5
Ileal carcinoid	Ileum (p)	No	<2
Ileal carcinoid	Muscle (m)	Yes	<2
Ileal carcinoid	Ileum (p)	Yes	<2
Ileal carcinoid	Ileum (p)	No	<2
Ileal carcinoid	Ileum (p)	Yes	<2
N-F pancreatic NET	Pancreas (p)	No	<2
N-F pancreatic NET	Pancreas (p)	No	5
Functioning pancreatico-duodenal NETs			
*Gastrinoma*	Retro-peritoneal LN (m)	Yes	5
*Glucagonoma*	Retro-peritoneal LN (m)	Yes	<2
*Glucagonoma*	pancreas (p)	Yes	5
*Glucagonoma*	Pancreas (p)	Yes	50
*Glucagonoma/Somatostatinoma*	Pancreas (p)	No	<2
*Somatostatinoma*	Pancreas (p)	Yes	<2
*Somatostatinoma*	Papilla Vatery (p)	No	<2
Pheochromocytoma	Adrenal medulla (p)	No	<2
Pheochromocytoma	Adrenal medulla (p)		<2
Pheochromocytoma	Adrenal medulla (m)	Yes	<2
Pheochromocytoma	Adrenal medulla (p)	No	10
Pheochromocytoma	Adrenal medulla (p)		<2
Pheochromocytoma	Adrenal medulla (p)	Yes	5
Pheochromocytoma	Adrenal medulla (p)	No	<2
Pheochromocytoma	Adrenal medulla (p)	No	25
Paraganglioma	Retroperitoneum (p)	No	<2
NE colon carcinoma	Colon (p)	Yes	50
NE colon carcinoma	Colon (p)	Yes	85
NE colon carcinoma	Colon (p)	Yes	<2
NE colon carcinoma	Liver (m)	Yes	5
Gastric NET, type 3	Corpus (p)	No	5

**^a^** Primary tumor (p) or metastasis (m) is indicated in parenthesis.

### 2.4. RNA Extraction and Reverse Transcription

Samples were weighted and Total RNA from 20–30 mg tissue of each preparation was extracted using the guanidinium thiocyanate-phenol-chloroform extraction method with TRI REAGENT^®^ (Molecular Research Center, Cincinnati, OH, USA). 

RNA integrity was examined by use of the Agilent 2100 Bioanalyzer^™^ (Agilent Technologies, Santa Clara, CA, USA) and RNA quantified by spectrophotometry by use of the Eppendorf Biophotometer^™^ (Eppendorf, Hamburg, Germany) or the Nanodrop^™^ ND-1000 spectrophotometer (Thermo Scientific, Wilmington, DE, USA).

Total RNA (0.5 µg) from each sample was reversed transcribed using Stratagenes AffinityScript^™^ QPCR cDNA Synthesis Kit (Stratagene, La Jolla, CA, USA) according to the protocol of the manufacturer. A mixture of random primers and Oligo(dT) primers (ratio 1:3) were used. The thermal protocol applied to all samples was 25 °C for 5 min, 42 °C for 15 min and 95 °C for 5 min.

### 2.5. Quantitative Real-Time PCR

Primers, probes as well as duplex (HK1 and β-actin) and triplex (GLUT1, HK2 and β-actin) assays were designed using Stratagene Laboratory Tools software (http://www.stratagene.com/qpcr/links.asp) [14], which was based on Primer 3 (http://primer3.sourceforge.net/) [15]. Probes were labeled with a fluorescent dye (FAM, HEX or CY5) and a quencher dye (Black-Hole-quencher 1 for FAM and HEX, and Black-Hole-quencher 2 for CY5). Primers (forward, FP and reverse, RP) as well as probes (Taq) were purchased from MWG (Ebersberg, Germany). 

Primer sequences were GLUT1-FP: 5’-CATCATCTTCATCCCGGC-3’, GLUT1-RP: 5’-CTCCTCGTTGCGGTTGAT-3’, HK1-FP: 5’-GGTGAAATCGTCCGCAAC-3’, HK1-RP: 5’-CCCGGGTCTTCATCGTC-3’, HK2-FP: 5’-CGGCCGTGCTACAATAGG-3’, HK2-RP: 5’-CTCGGGATCATGTGAGGG-3’, β-actin-FP: 5’-TGACAGCAGTCGGTTGGA-3’, β-actin-RP: 5’-CAAAGTCCTCGGCCACAT-3’. The probe sequences were GLUT1-Taq: 5’-AGTGCATCGTGCTGCCCTTCTG-3’, HK1-Taq: 5’-TCGACTTCACCAAGAAGGGATTCC-3’, HK2-Taq: 5’-TGCTTTAGACGTGTGACTGGGCAG-3’, β-actin-Taq: 5’-CGAGCATCCCCCAAAGTTCACA-3’.

Gene expression was quantified on the Mx3000p^™^ real-time PCR system (Stratagene, La Jolla, CA, USA) using core kit reagents (Stratagene) with a 50% increase (duplex assays) or a 100% increase (triplex assays) in dNTP and Taq polymerase concentration compared to the recommended concentration for simplex assays.

Aliquots of 25 µL in triplicate were made for all samples. One µL of cDNA was applied to each well. Standard curves with freshly made 5-fold dilutions were always included in the experiments for efficiency corrections. The thermal protocol applied to all assays was 10 min at 95 °C followed by 45 cycles with 30 s at 95 °C and 1 min at 60 °C. The ΔΔC_t_ method was used for calculation of the relative gene expression level as previously described [16]. 

### 2.6. Immunohistochemical Evaluation of Ki67

Formalin-fixed paraffin-embedded tissue samples were cut at 4 µm thick sections and mounted on coated slides. Antigens were retrieved with DAKO Target Retrieval Solution, High pH for 20 min at 97 °C (Code K8002) using the DAKO pre-treatment (PT) link. After blocking of endogenous peroxidase activity with Dako EnVision Flex+ (DAKO K8002) for 5 min, tissue sections were incubated with Monoclonal Mouse Anti-human Ki67 Antigen, DAKO Code M7240 at a dilution of 1:200 for 20 min at room temperature.

The reaction was visualized by using DAKO EnVision Flex+ mouselink for 15 min followed by DAKO EnVision Flex+/HRP for 20 min and finally DAKO EnVision Flex+ diaminobenzidine (DAB) for 10 min. The sections were counterstained with hematoxylin for 1 min. A section of the human tonsil was used as a positive control. Reading of slides was performed by counting positive tumor nuclei per 100 tumor cells.

### 2.7. Statistical Analysis

Comparison of categorical variables was performed using χ^2^ test. Comparison between continuous variables between the two groups was performed using a two-sided, unpaired *t*-test. Log transformation was performed on gene expression prior to testing to ensure normal distribution. Linear regression analysis was used for analysis of association between variables. *P* < 0.05 was considered significant.

## 3. Results

### 3.1. Gene Expression of GLUT, HK1 and HK2

Gene expression analysis was performed on all samples using quantitative real-time PCR and efficiency of the assays were in all cases between 91% and 106%. All three genes investigated, GLUT1, HK1, and HK2 as well as the normalizing gene β-actin were detected in all tumor and normal samples. Analysis of the assay reproducibility (by testing the same tissue sample 10 times) revealed that a 0.15-fold change in gene expression could be considered significant. Accordingly, a tumor:normal ratio of >1.15 was considered as upregulated. 

**Figure 1:**
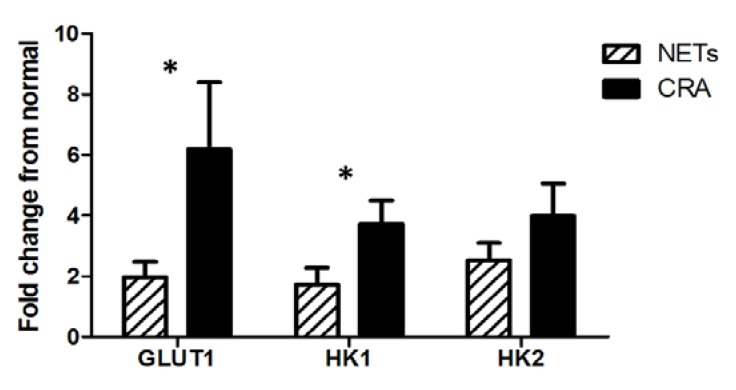
Gene expression level of glucose transporter 1 (GLUT1), and hexokinases (HK) HK1 and HK2 in the NET group (black bars) and the colorectal adenocarcinoma (CRA) group (grey bars). Error bars are expressed as S.E.M., (*) significant difference (*P* < 0.05) between NET and CRA group for the respective genes of interest.

FDG-PET identified malignant foci in significantly fewer NETs (4 of 11; 36%) than CRAs (6 of 7; 86%), χ^2^ test, *P* = 0.04 and significantly fewer NETs displayed upregulation of GLUT1 (13 of 34; 38%) compared to the CRA group (12 of 14; 86%) (χ^2^ test, *P* = 0.004). For HK1 there was no significant difference between the two groups, with HK1 being upregulated in 14 of 34 NETs (41%) compared to 10 of 14 CRAs (71%) (χ^2^ test, *P* = 0.111). Upregulation of HK2 gene expression did not differ significantly between the two groups (17 of 34 NETs; 50%, and 9 of 14 CRAs; 64%). When comparing the average gene expression level of the 3 genes between the NETs and the CRAs, the GLUT1 and HK1 gene expression levels were significantly lower in the NETs than the CRAs (GLUT1: *P* = 0.011, HK1: *P* = 0.049) whereas there was no significant difference in the HK2 gene expression level between the two groups (Figure 1). 

For the NETs there was a significant correlation between the gene expression level of GLUT1 and HK1 (R = 0.6, *P* = 0.001) whereas there was no significant correlation between GLUT1 and HK2. For the CRAs there was no significant correlation between GLUT1 and HK1 or between GLUT1 and HK2.

### 3.2. Ki67 and Correlation with Gene Expression of GLUT1, HK1 and HK2

**Figure 2:**
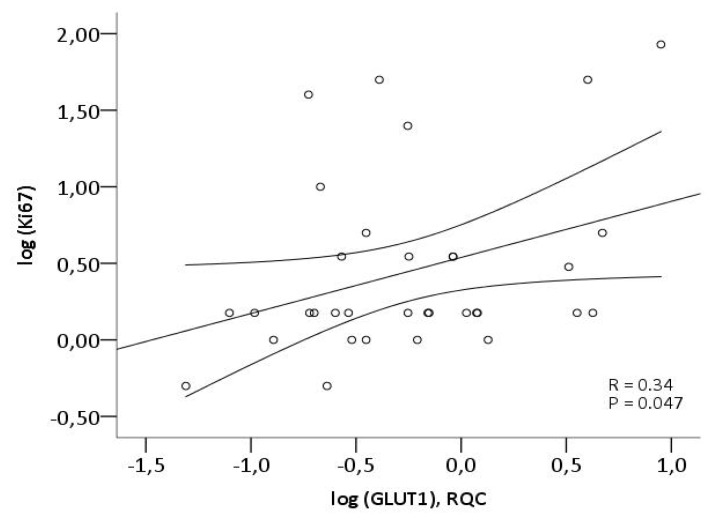
Correlation between GLUT1 gene expression and proliferation index in the NET group determined by qPCR and immunohistochemistry (IHC), respectively.

The proliferation index (Ki67) was determined by immunohistochemistry (IHC) in the NETs. As shown in Figure 2 there was a significant correlation between the Ki67 proliferation index of the NETs and the GLUT1 gene expression (R = 0.34, *P* = 0.047) (Figure 2), whereas no significant correlation between Ki67 index and HK1 or HK2 gene expression was found. By multivariate analysis with GLUT1, HK1 and HK2 in the model the results were similar, with only GLUT1 remaining in the model as independent predictor of proliferative rate as determined by Ki67 index.

### 3.3. FDG-PET and Gene Expression of GLUT1, HK1 and HK2

FDG-PET scans were performed in 11 of the patients in the NET group and 7 in the CRA group prior to resection. The FDG-PET scan was positive in 4 of the 11 NETs (36%) with a maximal standard uptake value (SUVmax) between 6.2 and 15.4. GLUT1 was upregulated in 3 of the 4 NETs with a positive FDG-PET (1.2–9-fold upregulation), HK1 in 2 of 4 (both 4-fold upregulation) and HK2 in all 4 (1.5–5-fold upregulation), see Table 2. In the CRA tumor group, 6 of the 7 FDG-PET scans were positive. The SUVmax of the tumors on the 6 positive scans were 3.8–34.8. All 6 patients with positive scans displayed upregulation of GLUT1 (1.7–33-fold upregulation), whereas the gene expression of HK1 and HK2 was upregulated in 4 of the 6 patients with positive FDG-PET scans (1.6–7 and 5–15-fold upregulation, respectively). 

For the CRAs (n = 7) upregulation of GLUT1 was significantly associated with a positive FDG-PET scan (χ^2^ test, *P* = 0.008). Neither HK1 nor HK2 were associated with a positive FDG-PET scan. For the NETs (n = 11), the only factor associated with a positive FDG result was a Ki67 proliferation index above 15 % (χ^2^ test, *P* = 0.04). The average SUVmax value for the FDG-PET positive cases was 9.4 for NET group (n = 4) and 11.9 for the CRA group (n = 6). 

**Table 2:** 18F-Fluorodeoxyglucose-positron emission tomography (FDG-PET) results, gene expression and proliferation index in the NET group. The gene expression level is expressed as the level in the tumor relative to the level in the non-cancerous tissue.

	FDG-PET result	GLUT1 gene expression	HK1 gene expression	HK2 gene expression	Ki67 index
Ileal carcinoid	Neg	5	1.1	1.1	5
Ileal carcinoid	Neg	4	3	1.8	<2
Ileal carcinoid	Neg	1.3	0.4	1.5	<2
Ileal carcinoid	Neg	1.1	0.8	1.6	<2
Pancreatico-duedenal NET *(Somatostatinoma)*	Neg	0.4	1.5	4	<2
Pancreatico-duedenal NET *(glucagonoma)*	Pos	0.9	0.1	1.5	2–5
Pancreatico-duedenal NET *(non-functioning)*	Pos	1.2	0.5	1.5	<2
NE colon carcinoma	Pos	4	4	3	50
NE colon carcinoma	Pos	9	4	5	85
NE colon carcinoma	Neg	0.6	1.6	1.7	2–5
NE colon carcinoma	Neg	0.3	1.7	4	<2

## 4. Discussion

Patients with NETs generally have a good prognosis compared to other cancer entities, with a reported overall 5-year survival rate of approximately 90% for gastrointestinal NETs compared to an overall survival rate of 25%–65% for gastrointestinal adenocarcinomas [17,18]. The same differences exist between pancreatic NETs and adenocarcinomas, with reported overall survival rates of 65%–70% [19,20] and 5%–20% [18,21,22], respectively. Since an energy shift towards increased glycolysis is seen in cancer cells reflecting the malignancy of the cells, we hypothesized that NETs have a lower glycolytic activity than the generally more malignant adenocarcinomas reflected in a lower expression level of glycolysis associated genes. Based on this, we investigated the gene expression of the glycolysis-associated genes GLUT1, HK1 and HK2 in NETs in comparison with a group of liver metastases from CRAs. We found that the gene expression level of GLUT1 and HK1 was significantly lower in the NET group than the CRA group, confirming our hypothesis. 

When available, we also compared the gene expression results with imaging results of FDG-PET scans as well as the proliferation rate of the tumor for a more thorough understanding of the gene expression results obtained. FDG-PET imaging, which visualizes glycolytic activity, is the most widely used nuclear medicine imaging modality applied in cancer diagnostics, staging of disease, and recurrence evaluation [23,24,25,26]. We have recently shown that despite the lower aggressiveness of NETs, reflected in a low proliferation index, FDG-PET has a high diagnostic sensitivity for WHO class III NETs [4], and can predict survival of NETs regardless of WHO grade [27].

In the present study, we found that the over-expression of the three glycolysis associated genes, GLUT1, HK1, and HK2, was not consistently seen in the NETs, which confirms the lower glucose utilization of NETs compared to several other cancer entities. Thus, only in 38% of the NETs investigated, GLUT1 gene expression was increased, which was significantly less than the CRAs where 86% of the tumors had increased GLUT1 gene expression. However, in the 38% of NETs where upregulation of GLUT1 was found, the gene expression level corresponded to the level in the CRA tumor group, and FDG-PET imaging may still be relevant for these patients. Also confirmed by the average SUVmax values of the two tumor groups, which did not differ significantly, some of the NETs are indeed aggressive neoplasms. For the CRAs, the GLUT1 gene expression and FDG-PET seemed to correlate well, with GLUT1 being upregulated in 86% (12 of 14) and FDG-PET also being positive in 86% of the patients undergoing FDG-PET imaging (6 of 7 patients). 

A variable gene expression of the hexokinases was observed for the NETs as well as the CRAs. For the NETs, a relatively strong correlation between the HK1 and GLUT1 gene expression was found, which could not be found between the HK2 and GLUT1 gene expression. For the CRAs, the GLUT1 gene expression did not correlate with either of the two HKs, confirming what has been found by others, that the HK gene expression is variable in several cancer entities [28,29,30,31]. 

We have previously shown that the diagnostic sensitivity of FDG-PET was higher with increasing proliferation index from 41% in grade I NETs to 92% in WHO grade III NETs [4]. Another recent study also found a strong correlation between proliferation index and FDG-PET result confirming the higher sensitivity for the more aggressive tumors [32]. Since determination of the proliferation index of the tumor is crucial for grading of NETs [2,33] and thereof choice of treatment, FDG-PET could perhaps be used as a non-invasive supplement to the pathological examination for grading of NETs. In the present study, we also found that a Ki67 index above 20% was associated with a positive FDG-PET scan. In addition, we found a significant but modest correlation between Ki67 index and GLUT1 gene expression in the NETs, indicating that the aggressiveness of the tumor is to some degree reflected in the GLUT1 expression.

It is important to bear in mind that FDG accumulation is a rather unspecific mechanism occurring in all tissue with increased glucose consumption. Therefore, it is not surprising that proliferative state and the glycolysis associated genes GLUT1, HK1 and HK2 cannot alone explain the FDG uptake in the tumor tissue. Several other factors, such as the hypoxic state of the tumor are also of relevance. Despite the modest correlation of the glycolysis associated genes with the proliferation index and the FDG-PET result, the present study show that the overall lower malignancy and accordingly better prognosis of NETs is reflected in a lower level of expression of glycolysis associated genes compared to the more aggressive CRAs. 

Several recent reports have elucidated that proliferation index alone cannot explain the aggressiveness of the tumor. Especially the discrimination between WHO grade I and II is troublesome. Assessment of several biomarkers of aggressiveness instead of just one could perhaps be of benefit for a more accurate grading and ultimately perhaps treatment selection. From our preliminary results GLUT1 gene expression could potentially be one such marker with added information regarding the aggressive potential of the tumors. 

## 5. Conclusions

GLUT1 gene expression was less frequently upregulated in NETs, compared to CRAs. A positive but modest correlation was found between GLUT1 and the Ki67 index, whereas HK1 and HK2 did not correlate with Ki67. FDG reflected GLUT1 gene expression in CRA and most CRA were FDG positive whereas less than half of the NETs were positive. 
